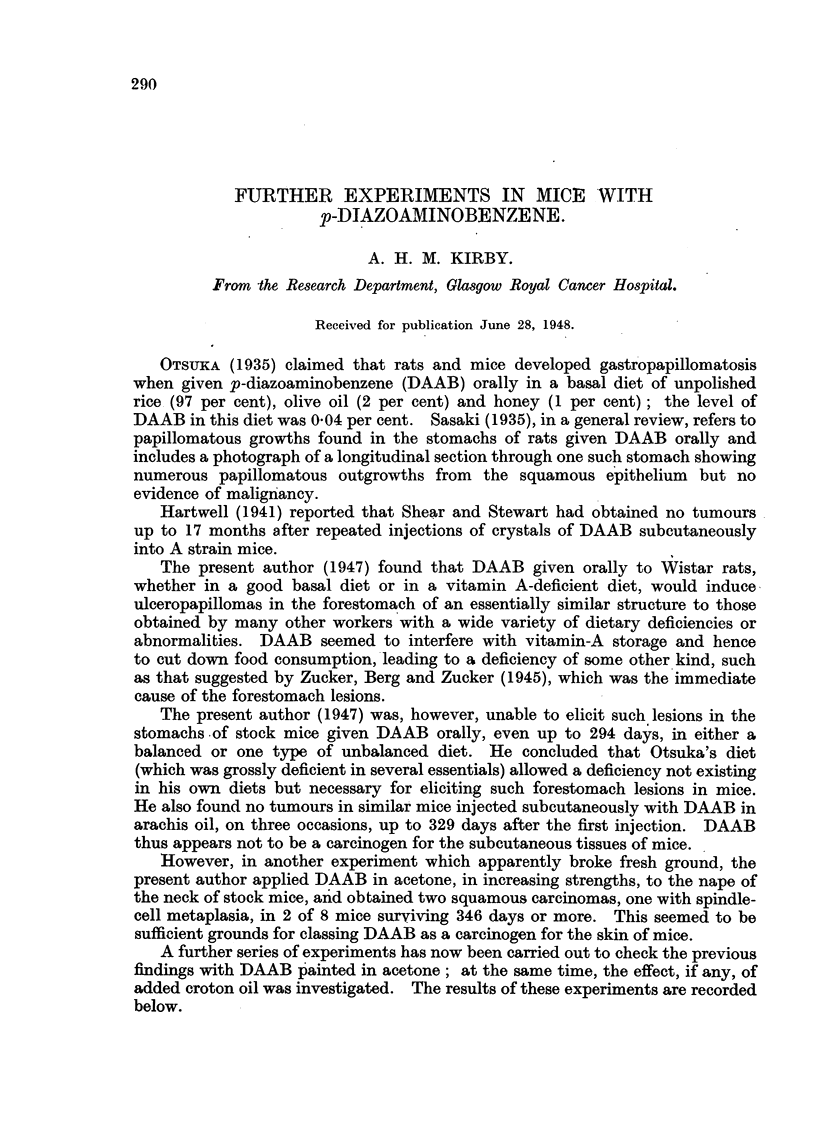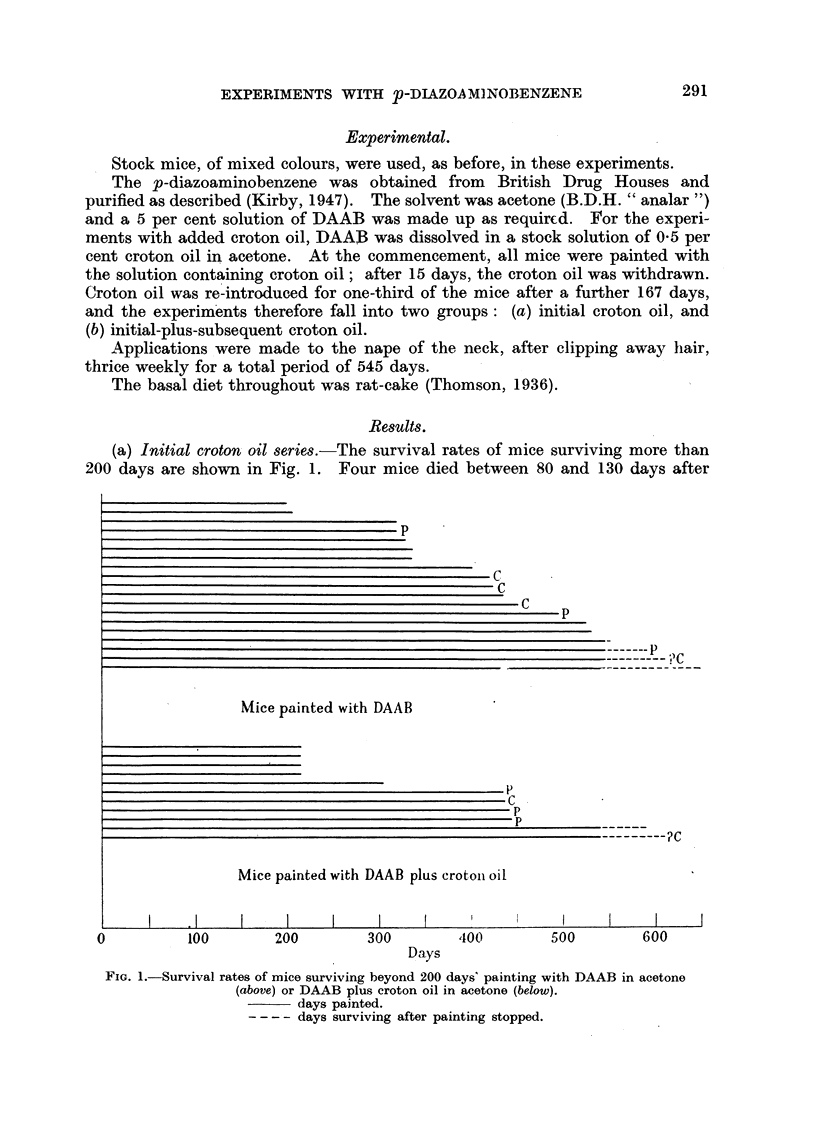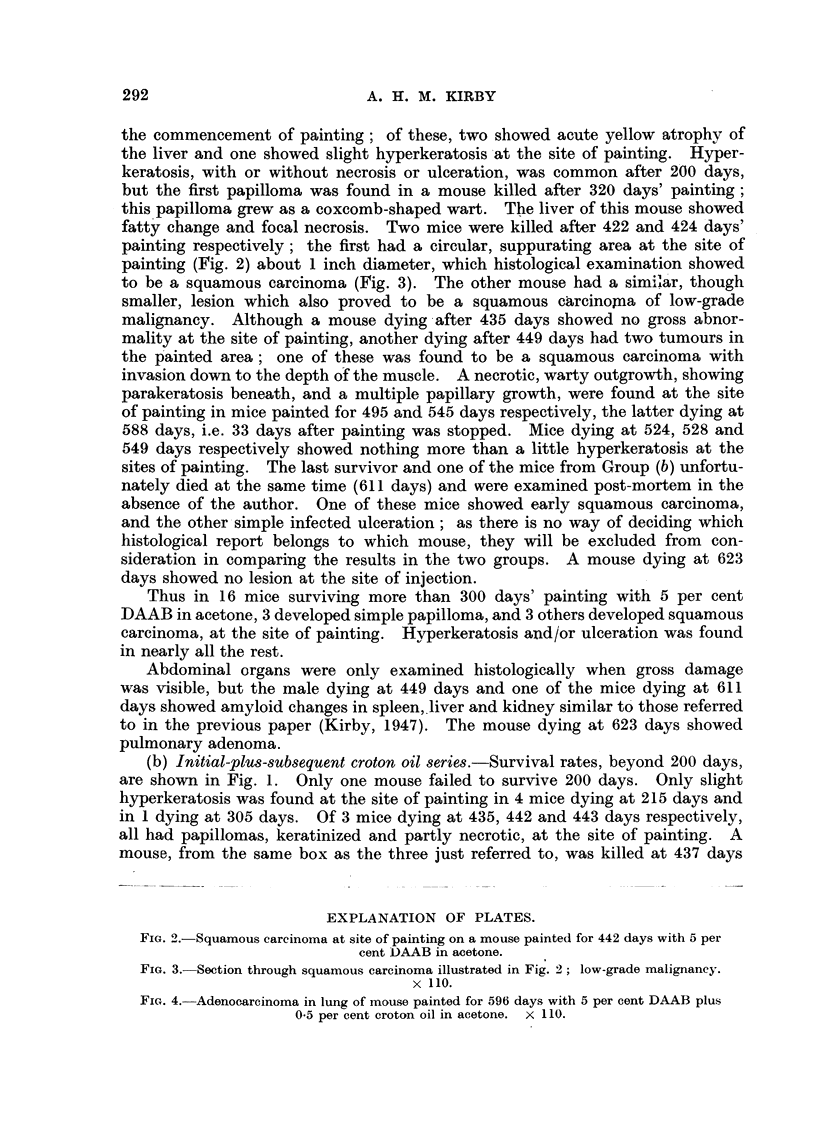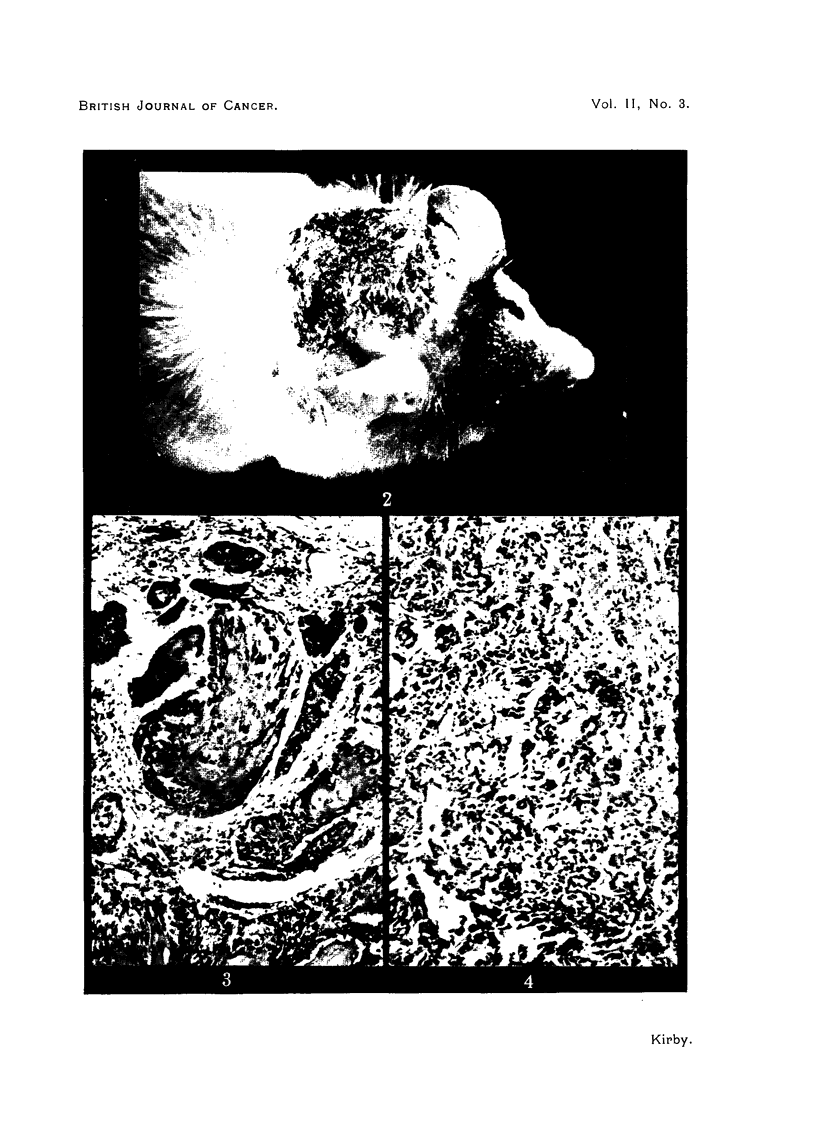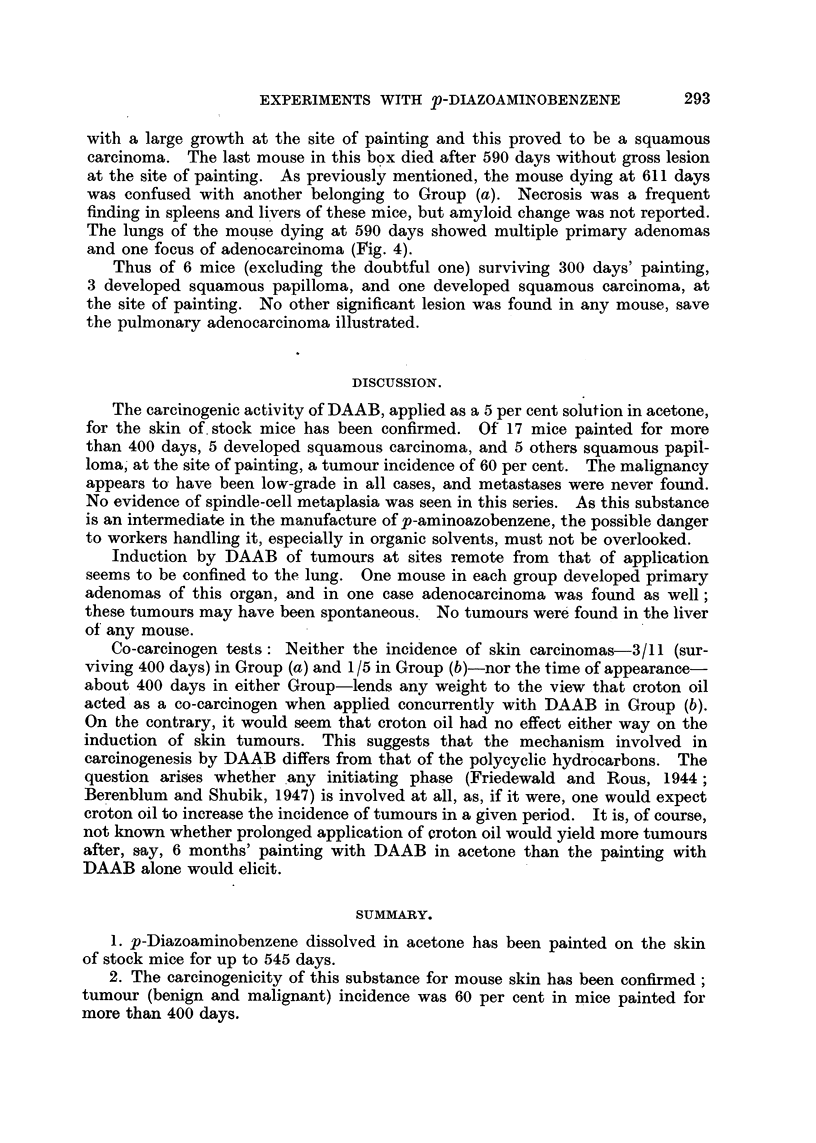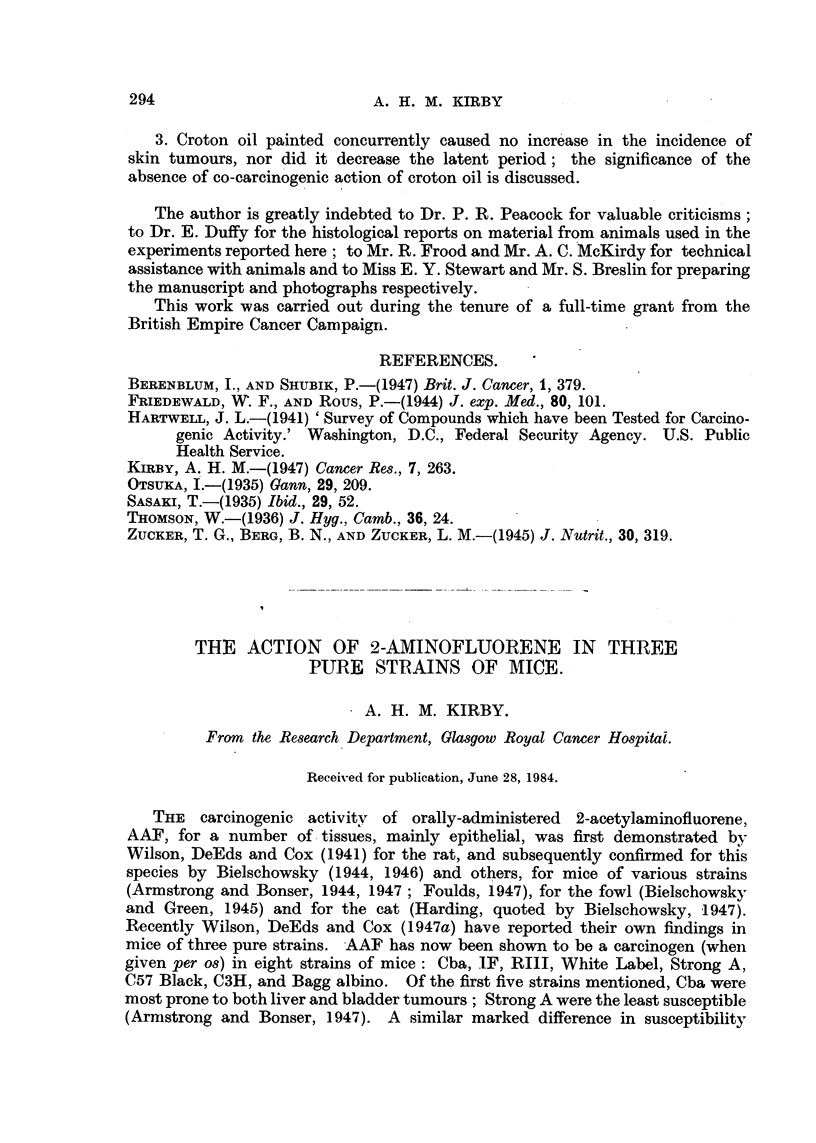# Further Experiments in Mice with p-Diazoaminobenzene

**DOI:** 10.1038/bjc.1948.34

**Published:** 1948-09

**Authors:** A. H. M. Kirby

## Abstract

**Images:**


### FURTHER EXPERIMENTS IN MICE WITH *p*-DIAZOAMINOBENZENE.

A. H. M. KIRBY.

*From the Research Department, Glasgow Royal Cancer Hospital.*

Received for publication June 28, 1948.

OTSUKA (1935) claimed that rats and mice developed gastropapillomatosis when given *p*-diazoaminobenzene (DAAB) orally in a basal diet of unpolished rice (97 per cent), olive oil (2 per cent) and honey (1 per cent); the level of DAAB in this diet was 0·04 per cent. Sasaki (1935), in a general review, refers to papillomatous growths found in the stomachs of rats given DAAB orally and includes a photograph of a longitudinal section through one such stomach showing numerous papillomatous outgrowths from the squamous epithelium but no evidence of malignancy.

Hartwell (1941) reported that Shear and Stewart had obtained no tumours up to 17 months after repeated injections of crystals of DAAB subcutaneously into A strain mice.

The present author (1947) found that DAAB given orally to Wistar rats, whether in a good basal diet or in a vitamin A-deficient diet, would induce ulceropapillomas in the forestomach of an essentially similar structure to those obtained by many other workers with a wide variety of dietary deficiencies or abnormalities. DAAB seemed to interfere with vitamin-A storage and hence to cut down food consumption, leading to a deficiency of some other kind, such as that suggested by Zucker, Berg and Zucker (1945), which was the immediate cause of the forestomach lesions.

The present author (1947) was, however, unable to elicit such lesions in the stomachs of stock mice given DAAB orally, even up to 294 days, in either a balanced or one type of unbalanced diet. He concluded that Otsuka's diet (which was grossly deficient in several essentials) allowed a deficiency not existing in his own diets but necessary for eliciting such forestomach lesions in mice. He also found no tumours in similar mice injected subcutaneously with DAAB in arachis oil, on three occasions, up to 329 days after the first injection. DAAB thus appears not to be a carcinogen for the subcutaneous tissues of mice.

However, in another experiment which apparently broke fresh ground, the present author applied DAAB in acetone, in increasing strengths, to the nape of the neck of stock mice, and obtained two squamous carcinomas, one with spindle-cell metaplasia, in 2 of 8 mice surviving 346 days or more. This seemed to be sufficient grounds for classing DAAB as a carcinogen for the skin of mice.

A further series of experiments has now been carried out to check the previous findings with DAAB painted in acetone; at the same time, the effect, if any, of added croton oil was investigated. The results of these experiments are recorded below.

*Experimental.*

Stock mice, of mixed colours, were used, as before, in these experiments.

The *p*-diazoaminobenzene was obtained from British Drug Houses and purified as described (Kirby, 1947). The solvent was acetone (B.D.H. " analar ") and a 5 per cent solution of DAAB was made up as required. For the experiments with added croton oil, DAAB was dissolved in a stock solution of 0·5 per cent croton oil in acetone. At the commencement, all mice were painted with the solution containing croton oil; after 15 days, the croton oil was withdrawn. Croton oil was re-introduced for one-third of the mice after a further 167 days, and the experiments therefore fall into two groups : (*a*) initial croton oil, and (*b*) initial-plus-subsequent croton oil.

Applications were made to the nape of the neck, after clipping away hair, thrice weekly for a total period of 545 days.

The basal diet throughout was rat-cake (Thomson, 1936).

*Results.*

(a) *Initial croton oil series.*—The survival rates of mice surviving more than 200 days are shown in Fig. 1. Four mice died between 80 and 130 days after

FIG. 1.—Survival rates of mice surviving beyond 200 days' painting with DAAB in acetone (*above*) or DAAB plus croton oil in acetone (*below*).
——— days painted.
– – – – days surviving after painting stopped.

the commencement of painting ; of these, two showed acute yellow atrophy of the liver and one showed slight hyperkeratosis at the site of painting. Hyperkeratosis, with or without necrosis or ulceration, was common after 200 days, but the first papilloma was found in a mouse killed after 320 days' painting ; this papilloma grew as a coxcomb-shaped wart. The liver of this mouse showed fatty change and focal necrosis. Two mice were killed after 422 and 424 days' painting respectively ; the first had a circular, suppurating area at the site of painting (Fig. 2) about 1 inch diameter, which histological examination showed to be a squamous carcinoma (Fig. 3). The other mouse had a similar, though smaller, lesion which also proved to be a squamous carcinoma of low-grade malignancy. Although a mouse dying after 435 days showed no gross abnormality at the site of painting, another dying after 449 days had two tumours in the painted area ; one of these was found to be a squamous carcinoma with invasion down to the depth of the muscle. A necrotic, warty outgrowth, showing parakeratosis beneath, and a multiple papillary growth, were found at the site of painting in mice painted for 495 and 545 days respectively, the latter dying at 588 days, i.e. 33 days after painting was stopped. Mice dying at 524, 528 and 549 days respectively showed nothing more than a little hyperkeratosis at the sites of painting. The last survivor and one of the mice from Group (*b*) unfortunately died at the same time (611 days) and were examined post-mortem in the absence of the author. One of these mice showed early squamous carcinoma, and the other simple infected ulceration ; as there is no way of deciding which histological report belongs to which mouse, they will be excluded from consideration in comparing the results in the two groups. A mouse dying at 623 days showed no lesion at the site of injection.

Thus in 16 mice surviving more than 300 days' painting with 5 per cent DAAB in acetone, 3 developed simple papilloma, and 3 others developed squamous carcinoma, at the site of painting. Hyperkeratosis and/or ulceration was found in nearly all the rest.

Abdominal organs were only examined histologically when gross damage was visible, but the male dying at 449 days and one of the mice dying at 611 days showed amyloid changes in spleen, liver and kidney similar to those referred to in the previous paper (Kirby, 1947). The mouse dying at 623 days showed pulmonary adenoma.

(b) *Initial-plus-subsequent croton oil series.*—Survival rates, beyond 200 days, are shown in Fig. 1. Only one mouse failed to survive 200 days. Only slight hyperkeratosis was found at the site of painting in 4 mice dying at 215 days and in 1 dying at 305 days. Of 3 mice dying at 435, 442 and 443 days respectively, all had papillomas, keratinized and partly necrotic, at the site of painting. A mouse, from the same box as the three just referred to, was killed at 437 days

EXPLANATION OF PLATES.

FIG. 2.—Squamous carcinoma at site of painting on a mouse painted for 442 days with 5 per cent DAAB in acetone.

FIG. 3.—Section through squamous carcinoma illustrated in Fig. 2 ; low-grade malignancy. × 110.

FIG. 4.—Adenocarcinoma in lung of mouse painted for 596 days with 5 per cent DAAB plus 0·5 per cent croton oil in acetone. × 110.

2

3

4

with a large growth at the site of painting and this proved to be a squamous carcinoma. The last mouse in this box died after 590 days without gross lesion at the site of painting. As previously mentioned, the mouse dying at 611 days was confused with another belonging to Group (*a*). Necrosis was a frequent finding in spleens and livers of these mice, but amyloid change was not reported. The lungs of the mouse dying at 590 days showed multiple primary adenomas and one focus of adenocarcinoma (Fig. 4).

Thus of 6 mice (excluding the doubtful one) surviving 300 days' painting, 3 developed squamous papilloma, and one developed squamous carcinoma, at the site of painting. No other significant lesion was found in any mouse, save the pulmonary adenocarcinoma illustrated.

### DISCUSSION.

The carcinogenic activity of DAAB, applied as a 5 per cent solution in acetone, for the skin of stock mice has been confirmed. Of 17 mice painted for more than 400 days, 5 developed squamous carcinoma, and 5 others squamous papilloma, at the site of painting, a tumour incidence of 60 per cent. The malignancy appears to have been low-grade in all cases, and metastases were never found. No evidence of spindle-cell metaplasia was seen in this series. As this substance is an intermediate in the manufacture of $p$-aminoazobenzene, the possible danger to workers handling it, especially in organic solvents, must not be overlooked.

Induction by DAAB of tumours at sites remote from that of application seems to be confined to the lung. One mouse in each group developed primary adenomas of this organ, and in one case adenocarcinoma was found as well; these tumours may have been spontaneous. No tumours were found in the liver of any mouse.

Co-carcinogen tests: Neither the incidence of skin carcinomas—3/11 (surviving 400 days) in Group (*a*) and 1/5 in Group (*b*)—nor the time of appearance—about 400 days in either Group—lends any weight to the view that croton oil acted as a co-carcinogen when applied concurrently with DAAB in Group (*b*). On the contrary, it would seem that croton oil had no effect either way on the induction of skin tumours. This suggests that the mechanism involved in carcinogenesis by DAAB differs from that of the polycyclic hydrocarbons. The question arises whether any initiating phase (Friedewald and Rous, 1944; Berenblum and Shubik, 1947) is involved at all, as, if it were, one would expect croton oil to increase the incidence of tumours in a given period. It is, of course, not known whether prolonged application of croton oil would yield more tumours after, say, 6 months' painting with DAAB in acetone than the painting with DAAB alone would elicit.

### SUMMARY.

1. $p$-Diazoaminobenzene dissolved in acetone has been painted on the skin of stock mice for up to 545 days.

2. The carcinogenicity of this substance for mouse skin has been confirmed; tumour (benign and malignant) incidence was 60 per cent in mice painted for more than 400 days.

3. Croton oil painted concurrently caused no increase in the incidence of skin tumours, nor did it decrease the latent period ; the significance of the absence of co-carcinogenic action of croton oil is discussed.

The author is greatly indebted to Dr. P. R. Peacock for valuable criticisms ; to Dr. E. Duffy for the histological reports on material from animals used in the experiments reported here ; to Mr. R. Frood and Mr. A. C. McKirdy for technical assistance with animals and to Miss E. Y. Stewart and Mr. S. Breslin for preparing the manuscript and photographs respectively.

This work was carried out during the tenure of a full-time grant from the British Empire Cancer Campaign.

### REFERENCES.

Berenblum, I., and Shubik, P.—(1947) *Brit. J. Cancer*, **1**, 379.
Friedewald, W. F., and Rous, P.—(1944) *J. exp. Med.*, **80**, 101.
Hartwell, J. L.—(1941) 'Survey of Compounds which have been Tested for Carcinogenic Activity.' Washington, D.C., Federal Security Agency. U.S. Public Health Service.
Kirby, A. H. M.—(1947) *Cancer Res.*, **7**, 263.
Otsuka, I.—(1935) *Gann*, **29**, 209.
Sasaki, T.—(1935) *Ibid.*, **29**, 52.
Thomson, W.—(1936) *J. Hyg., Camb.*, **36**, 24.
Zucker, T. G., Berg, B. N., and Zucker, L. M.—(1945) *J. Nutrit.*, **30**, 319.